# Mesh trimming and suture reconstruction for wound dehiscence after huge abdominal intercostal hernia repair: A case report

**DOI:** 10.1016/j.ijscr.2018.11.028

**Published:** 2018-11-22

**Authors:** Yuta Takeuchi, Yo Kurashima, Yoshitsugu Nakanishi, Toshimichi Asano, Takehiro Noji, Yuma Ebihara, Soichi Murakami, Toru Nakamura, Takahiro Tsuchikawa, Keisuke Okamura, Toshiaki Shichinohe, Satoshi Hirano

**Affiliations:** Department of Gastroenterological Surgery II, Hokkaido University Faculty School of Medicine, North 15 West 7, Kita-ku, Sapporo 0608638, Hokkaido, Japan

**Keywords:** Abdominal intercostal hernia, Mesh exposure, Wound dehiscence

## Abstract

**Introduction:**

Abdominal intercostal hernia repair for huge incisional hernia after thoracoabdominal surgery involves a complex anatomical structure. Hence, it is difficult to apply the laparoscopic approach to large hernias in the lateral upper abdomen. Further the optimal approach to mesh exposure without infection after incisional hernia repair is still controversial. Herein, we describe our experience of repairing a huge abdominal intercostal hernia by mesh trimming and suture reconstruction for wound dehiscence.

**Presentation of case:**

A 73-year-old man presented with an incisional hernia in the left flank from just below the eight intercostal space to the transverse umbilical region 6 months after thoracoabdominal aortic aneurysm surgery. Computed tomography revealed an incisional hernia orifice of 17 × 13 cm located on the left flank around the ninth rib. We chose the open approach as treatment because the hernia orifice was large, and we created a mesh placement space in the extraperitoneal cavity and placed expanded polytetrafluoroethylene mesh there with 1–0 nonabsorbable monofilament suture. At postoperative day 26, we observed mesh exposure due to wound dehiscence. Mesh trimming and suture reconstruction for wound dehiscence was performed because there were no signs of wound infection. The postoperative course was uneventful including infection and dehiscence. The patient has been well without recurrence for 14 months since last operation.

**Conclusions:**

Optimal treatment for repair of a large abdominal intercostal hernia with thoracoabdominal location is necessary. Moreover, partial mesh removal may be one of the treatment options for mesh exposure if conditions are met.

## Highlights

• Large abdominal intercostal hernia in the thoracoabdominal region must be treated.
• Repair of abdominal intercostal hernia using mesh and surgical approach is controversial.
• With exposed mesh, partial mesh removal may be an option if conditions are met.

## 1 Introduction

Reports on abdominal intercostal hernia repair for huge incisional hernia after thoracoabdominal surgery is limited because it involves a complex anatomical structure [1]. Although the laparoscopic approach has been widely used for incisional hernia repair, it is still challenging to perform in large hernias located in the lateral upper abdomen. In addition, the optimal approach to mesh exposure without infection after incisional hernia repair is still controversial. We present our experience of repairing a huge abdominal intercostal hernia due to thoracoabdominal aortic aneurysm surgery by mesh trimming and suture reconstruction for wound dehiscence. This work has been reported in line with the SCARE criteria [2].

## 2 Presentation of case

**Fig. 1:**
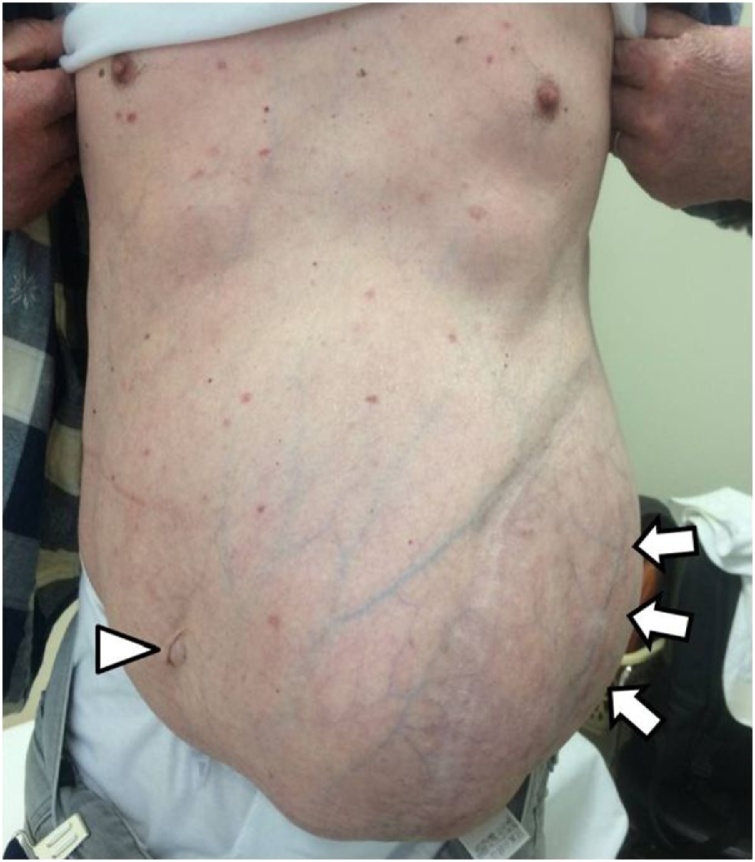
Physical examination: The umbilicus (arrowhead) was positioned toward the right side because of a mass. A reducible mass (arrow) at the left upper quadrant corresponding to about 30 cm.

**Fig. 2:**
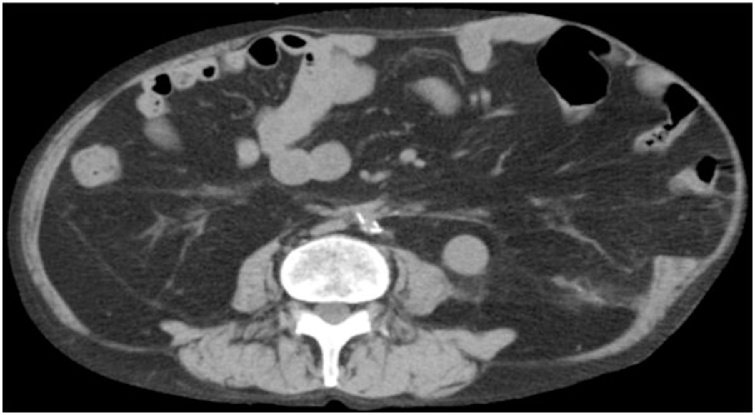
Computed tomography (CT) findings. CT revealed a 17 × 13 cm incisional hernia from the ninth rib to the left flank.

A 73-year-old man presented with an incisional hernia in the left flank from just below the eight intercostal space to the transverse umbilical region 6 months after thoracoabdominal aortic aneurysm surgery. While the progression was observed in the clinic, the hernia has grown bigger accompanied by worsening symptoms, such as walking difficulty. On physical examination (Fig. 1), the patient weighed 57.9 kg, has a height of 165 cm, and a body mass index of 21.3. He had a reducible mass at the left upper quadrant corresponding to about 30 cm. Reported comorbidities were diabetes and granulomatosis with polyangiitis treated with steroids and immunosuppressants. Computed tomography (CT) revealed a 17 × 13 cm incisional hernia orifice from the ninth rib to the left flank. The hernia contained the small intestines (Fig. 2). We planned surgery for the huge incisional hernia because his clinical symptoms had exacerbated.

The patient was placed on the right semi-lateral decubitus position under general anesthesia. A 20-cm long incision was made over the hernia along the previous wound. The hernia sac was identified and opened. There was no adhesion between the hernia sac and intra-abdominal contents. We tried to secure a space to place the mesh (35 × 25 cm in size) in the extraperitoneal space. The mesh was expanded polytetrafluoroethylene: GORE^®^ DUALMESH^®^ Patch. The extent of the dissection for the placement of the mesh was the dorsal side of the left rectus abdominis muscle on the median, dorsal side of the rectus abdominis muscle and transverse abdominis muscle on the caudal side, and the lateral latissimus dorsi muscle on the lateral side. However, we could not secure the cranial side for mesh placement because of strong adhesion of the costal cartilage dissected at the previous surgery. Thus, we secured the upper space between the skin and the ventral side of the eighth rib. An expanded polytetrafluoroethylene (ePTFE) hernia mesh was then placed to cover the hernia defect, and we sutured along the outer edge of the mesh and hernial gate and partly sutured directly to the ribs using 1-0 nonabsorbable monofilament suture.

**Fig. 3:**
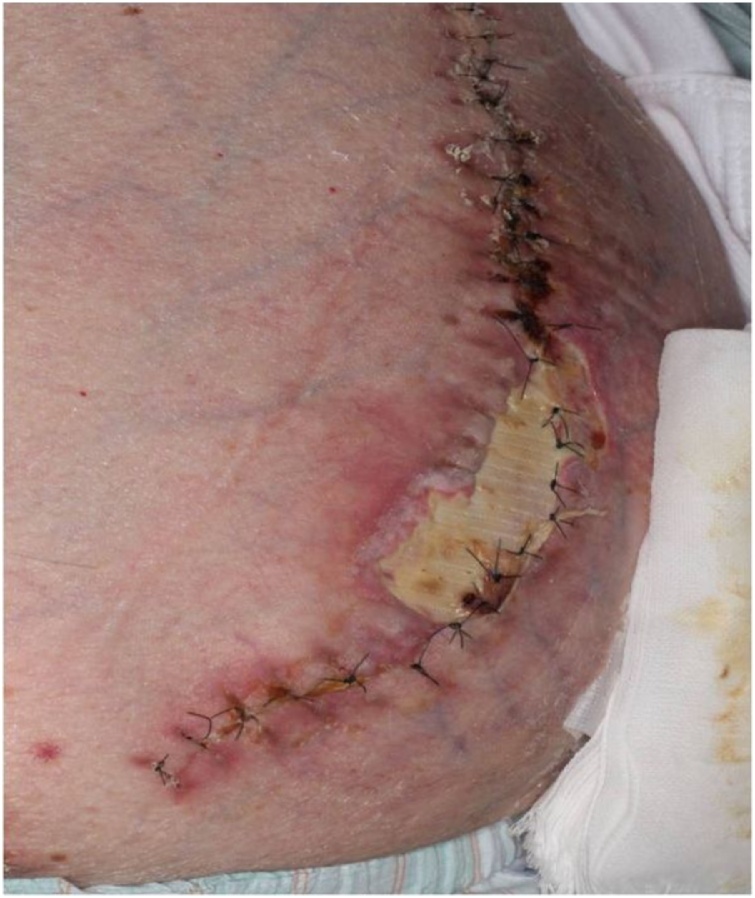
Postoperative day 26 wound findings: 3 × 7 cm mesh exposure due to wound dehiscence without infection.

**Fig. 4:**
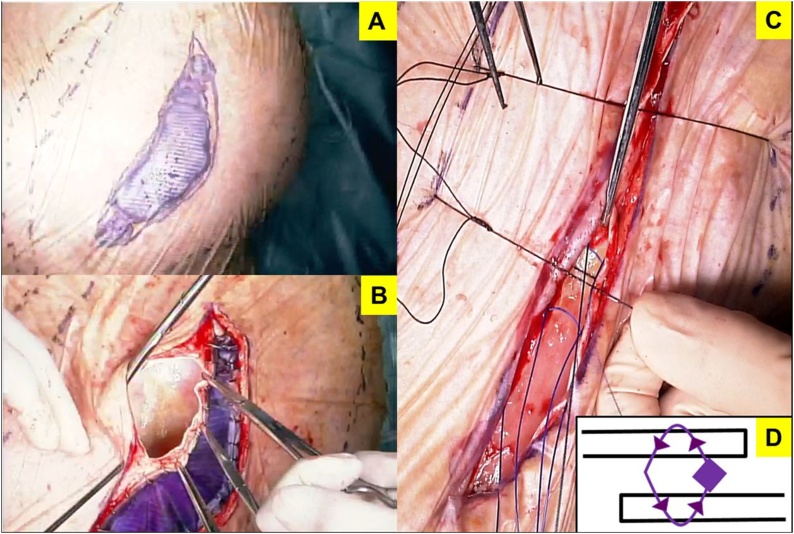
Re-operation procedure. (A) Put a crystal violet on the exposed mesh. (B) Local removal of the exposed mesh. Adhesion and abscess were not detected. (C) Overlapping the remaining mesh by 5 mm with 1-0 nonabsorbable suture and retention sutures in the skin. (D) Nodules concealed between meshes to prevent skin damage at the nodules.

**Fig. 5:**
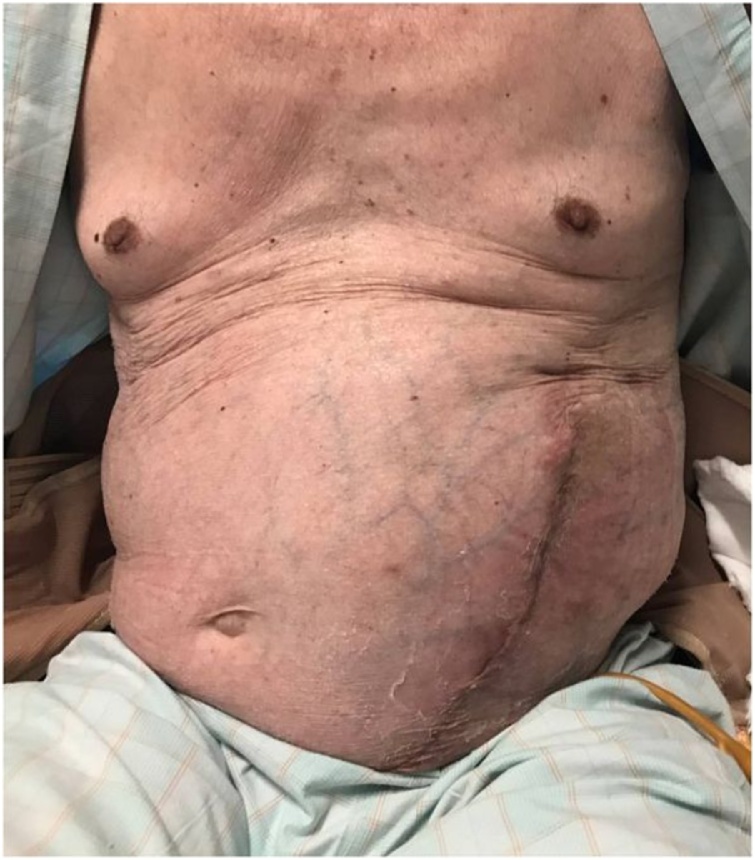
Final wound appearance. The wound is clear without infection and dehiscence at discharge.

On postoperative day 4, his general condition worsened due to aspiration pneumonia. Tracheotomy was performed for intensive whole-body management. Consequently, his general condition improved. On postoperative day 26, we detected that a 3 × 7 cm mesh was exposed due to wound dehiscence (Fig. 3). The findings of bacterial culture of this wound and the surface of the exposed mesh were negative. Initially, we had to choose a conservative management because of the poor condition of the patient. Although the general condition was improving, the wound dehiscence extended; hence, we performed mesh trimming and suture reconstruction (Fig. 4). While trimming the exposed mesh, adhesion and signs of infection, such as an abscess formation, were not observed. Both sides of the trimmed mesh were overlapped by 5 mm with nonabsorbable suture. The tied nodules concealed between meshes to prevent skin damage at the nodules. The skin was sutured using retention sutures to transfix the remaining mesh. The postoperative course was uneventful (Fig. 5). The patient has been well without recurrence for 14 months since the second operation.

## 3 Discussion

Incisional hernia is most commonly caused by abdominal surgery. Despite advances in the prevention and treatment of infection during surgery and use of some suture materials that have reduced the incidence of incisional hernias, incisional hernias still occur in 11%–20% after abdominal surgery [3,4].

Abdominal intercostal hernia in the lateral upper abdomen sometimes develops after thoracoabdominal surgery, nephrectomy, and trauma surgery associated with rib fracture [5]. This patient underwent thoracoabdominal surgery with the incision from abdomen to the eighth intercostal space. The incisional intercostal hernia was caused by the surgery that affected the intercostal muscles and diaphragm. Moreover, the patient has been treated with diabetes and was taking steroids and immunosuppressant for collagen disease.

The treatment for abdominal intercostal hernia is basically surgical repair to prevent the risk of incarceration and strangulation of the omentum, small intestine, or colon [1]. A summary of the recent reports on the treatment of abdominal intercostal hernia is shown in Table 1.

**Table 1:** Summary of reports on the treatment of abdominal intercostal hernia.

Case No.		Age	Sex	Prior surgery or cause	Hernia orifice size	Approach	Mesh type for hernia repair	Mesh placement
1	Abunnaja et al. [1]	51	Female	Stab injury	8 × 8 cm	Open	Polypropylene and ePTFE hernia patch	Underlay
2	Yamamoto et al. [5]	75	Female	Nephrectomy	Not described	Open	Polyester mesh coated with absorbent collagen	Intraperitoneal onlay
3	Benizri et al. [6]	66	Female	None	Not described	Open	Polypropylene mesh	Onlay
4	Benizri et al. [6]	83	Female	Trauma	Not described	Open	Polypropylene mesh	Intraperitoneal onlay＋Onlay
5	Akinduro et al. [13]	79	Male	Nephrectomy	2.5 cm	Open	Suture	Not
6	Bobbio et al. [14]	70	Male	Trauma	Not described	Laparoscopy	Ovaloid double mesh prosthesis	Intraperitoneal onlay
7	Rosch et al. [15]	57	Female	Nephrectomy	5 × 5 cm	Open	Polypropylene compound mesh	Underlay
8	Erdas et al. [16]	48	Male	Trauma	5.8 × 3 cm	Open	Polypropylene stiff mesh	Underlay
9	Gundara et al. [17]	88	Female	Minor trauma	3.5 cm	Laparoscopy	Composite mesh	Sublay
10	Geoffrey et al. [18]	28	Male	Trauma	7 × 8 cm	Laparoscopy	Suture	Not
Our Case	Our case	73	Male	Thoracoabdominal aortic aneurysm surgery	17 × 13 cm	Open	ePTFE hernia patch	Onlay

ePTFE, expanded polytetrafluoroethylene.

Repair of abdominal intercostal hernia using mesh and surgical approach is controversial. However, there were cases of incisional abdominal intercostal hernia repair using mesh [5,6]. The surgical approaches are open abdominal approach and laparoscopic approach [1]. In emergency cases of incisional hernia, the open abdominal approach is a safe operative choice to minimize the risk of intra-abdominal injuries [6]. Conversely, the laparoscopic approach provides a magnified view of the surgical field during surgery to confirm the contents of the hernia. However, if the hernia is huge, a larger mesh is required; therefore, surgery through the abdominal cavity is difficult. As shown in Table 1, mesh placement also varies. It is caused by the hernia involving a complex anatomical structure such as ribs or diaphragm. Hence, it is very difficult to fix a mesh appropriately.

Conservative management can be considered in some patients, particularly in asymptomatic cases and elderly patients with high surgical risk [1,6].

For our patient, we chose a direct approach because the hernia was large. Incisional hernia repair using a mesh has potential complications related to infection and wound dehiscence. Infection is the most common complication using a prosthetic mesh. Even if antibiotics are used, the infection rate is up to 1.5% [7]. Abdominal wound dehiscence is a serious postoperative complication with an incidence ranging from 0.4% to 1.2% [8]. However, mesh exposure without infection is rare. Hence, there has been little evidence about the management of exposed mesh after incisional hernia [9]. The conventional approach to mesh exposure is surgery with mesh removal or replacement. In choosing a conservative treatment, treatment with complex approach is necessary [8].

Maintenance of skin blood flow accelerates wound healing and prevents infection and mesh exposure due to wound dehiscence [9]. In our case, we consider that the causes of the exposed mesh were wound tension and poor blood circulation because of few subcutaneous fat tissues and a thin abdominal wall, as the patient was thin. In addition, there was delayed wound healing due to steroids and immunosuppressants.

When the exposed mesh on the wound dehiscence is found, the possibility of mesh infection should be considered. Thus, the priority treatment for infected mesh is removal [9]. On the contrary, mesh removal is associated with risks for acute bleeding, enterocutaneous fistula, and a larger incisional hernia due to failure to close the primary defect. Therefore, conservative treatment such as removal of skin sutures, opening of wound and drainage, irrigation with saline, and wound debridement are alternative treatments [7].

In this case, conservative treatment was first chosen, such as debridement and irrigation with saline, because of the patient’s poor condition. Since the wound dehiscence extended after improvement of his general condition, the dehiscence was then repaired. Luckily, the three wound cultures did not detect bacteria, and signs of infection were not observed intraoperatively. This case had no infection despite comorbidities with diabetes and granulomatosis with steroids and immunosuppressants. We assumed that it was influenced by mesh. The mesh was made of ePTFE. Its material has two surfaces: rough surface for tissue incorporation with micropores approximately about 22 mm and a smooth surface for minimal tissue attachment with micropores 3 mm [10]. Mesh with small pores (<10 μm) are considered to increase the risk of infection because of its permeability to bacteria, but not to neutrophils and macrophages [11]. However, mesh with large pore prevents infiltration and growth of bacteria, because it does not only admit macrophages, but also allows rapid fibroplasia and angiogenesis [12]. The fact that mesh infection did not develop may be affected by the large pore size of the mesh. If signs of infection, such abscess under the mesh, were found during the second operation, we would remove the mesh and choose another autologous treatment, such as tensor fascia lata musculocutaneous flap or split thickness skin grafting. Moreover, we were concerned that the defect would become larger if the entire mesh was removed, making wound closure difficult. Thus, only the exposed mesh was excised, and the trimmed mesh was overlapped to obtain a satisfactory outcome.

## 4 Conclusion

Optimal treatment for large abdominal intercostal hernia located in the thoracoabdominal region is necessary. When the mesh is exposed, partial mesh removal may be one of the treatment options if conditions are met.

## Conflict of interest

This research did not receive any specific grant from funding agencies in the public, commercial, or not-for-profit sectors.

## Sources of funding

The study sponsors had no such involvement.

## Ethical approval

The study is exempt from ethnical approval in my institution.

## Consent

Written informed consent was obtained from the patient for the publication of this report and any accompanying images at Hokkaido University Faculty School of Medicine.

## Author’s contributions

YT and YK drafted the manuscript. YN, TA, TN, YE, SM, TN, TT, KO, TS, and SH critically revised the manuscript.

## Registration of research studies

We performed informed consent fully and got consent from the patient.

## Guarantor

Yo Kurashima.

## Provenance and peer review

Not commissioned, externally peer reviewed.
